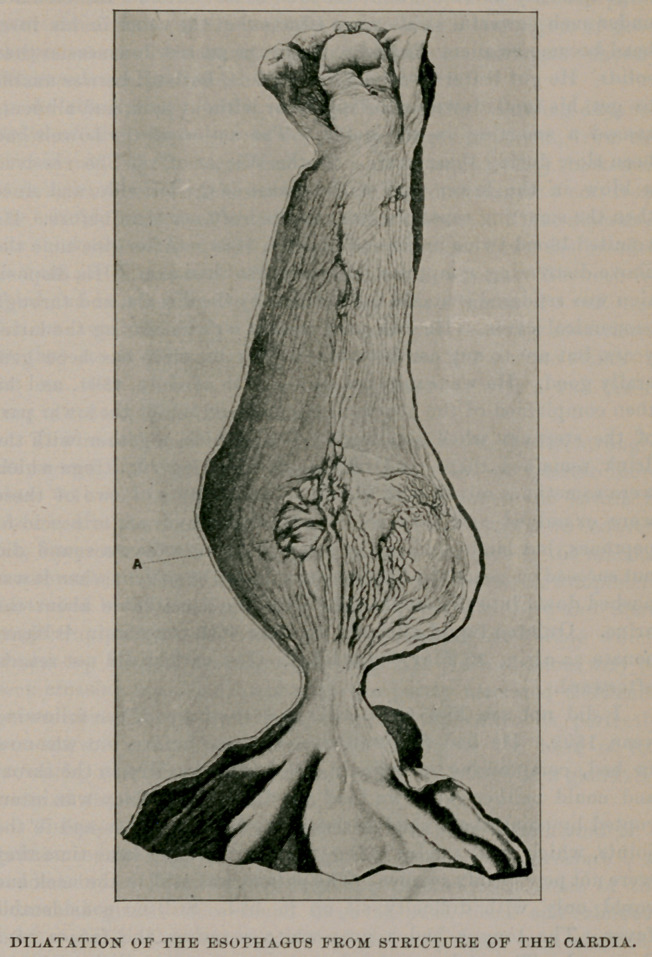# Dilatation of the Esophagus Caused by a Stricture in the Cardia

**Published:** 1896-02

**Authors:** E. G. Johnson


### Translation.

### DILATATION OF THE ESOPHAGUS CAUSED BY A STRICTURE IN THE CARDIA.

By DR. E. G. JOHNSON.

Condensed abstract from *Hygeia*, October, 1895.

Translated by L. G. SELLSTEDT, Buffalo.

IN THE first case described, the details in support of neurotic etiology may be comprehended under the following heads : improper food, irregular meals, deprivations in a rigid climate and nervous strain due to the duties of the position of the patient, a young clergyman, who, at the age of twenty-six, became troubled with intercostal rheumatism with swelling of the left hand. This continued till he was thirty years of age. During this time he also had frequent pains in the region of the left jaw with accompanying flow of saliva. At last an abscess formed without serious consequences. In 1892, his duties compelled many journeys under privations in Lapland and in February, 1893, he became aware of a difficulty in swallowing, so that it was necessary to wash down his solid food with liquids. Soon after he was taken with severe colic-like pains in the cardiac region, which the following year recurred with intervals of days or weeks. These attacks came on at night, generally, after a while in the latter part. They usually began with an itching sensation in the region of the xiphoid process, which soon changed into boring pains and as if pinched with tongs. The pains spread to the lower part of the thorax and he became tender to the touch over the inferior part of the sternum. Occasionally, especially from the close of 1893, these pains even invaded the throat and angles of the jaws, the posterior portion of the tongue and the walls of the larynx. They were sometimes absent if at the beginning of the paroxysm he shifted his position from left to right. They were oftenest mitigated by drinking cold water.

About this time both solid and liquid aliments would lodge in the esophagus, causing distress and burning sensations and, in spite of all efforts to swallow, both meat and drink would often come up unchanged, though he sometimes succeeded in getting food into the stomach after drinking. With these swallowing movements he experienced a bursting sensation in the chest, till the food had found its way into the stomach. Sometimes the food remained several hours in the gullet, but at such times the patient after a while was enabled to relieve himself by vomiting, the contents being more or less mingled with mucus. Whether the food remained in the esophagus or was ejected, he felt vertigo and uncertainty in his walk. Generally, the difficulty of deglutition was greatest at the beginning of a meal; if it succeeded then all would go well. The difficulty of swallowing returned at all kinds of troubles and cares, as when he feared the food would stick fast, when he felt himself observed, and the like. The evil was aggravated by public meetings, at church services, marriages, and the like, when he was compelled to extemporise. The irritation of the esophagus by the retention of aliments soon brought on severe coughing spells at night, which were only relieved by vomitings, which he felt were wholly from the esophagus. After this a drink of water would produce calm and sleep. Iodide of potash and vichy were given for the cough, and for the pain in the gullet, iron arsenic, quinine and opium with good effect. Lately, the patient has tried to evacuate the gullet before meals to aid the passage of food into the stomach. The urine has always been dark with red sediment. The patient has never had lues, has committed no excesses and never has used ardent spirits to excess. The 8th of July, 1895, a stomach tube 1.9 cm. in diameter was introduced and an obstruction was found 49 cm. below the dental range. This obstruction, after some seconds of gentle pressure, gave away so that the tube during a few moments passed through a rather narrow channel to go farther without hinder. The place where the sound stopped was directly under the point of the xiphoidal process. A tube of 1.5 cm. found the obstruction 46.5 cm. below the teeth and then, after a moment's gentle pressure, slid through the constricted place. No pain was felt by the insertion of the instrument.

From July 22d electricity was used upon the patient. After he had drank a glass of water an electric sound was carried down to the cardia and put into communication with the galvanic current. The opposite pole was applied by turns, to the left of the vertebral column, between the sixth and eighth ribs, to the pit of his stomach or the sternum in seances of from ten to fifteen minutes. Ronsegno and Vichy water was ordered for the patient, who, since the 8th of July, had taken forty grains of bromide of soda daily.

Present state, August 2d and the following days : patient's intellect is clear, memory and cheerfulness unimpaired ; he measures six feet, weighs 195 pounds, has a powerful frame, slender, normal adipose painculus, firm muscles, skin of normal appearance, with usual sensibility to touch or cold and heat, feels stiff about the eyes when he awakens, all his senses normal, the tongue exhibits throughout the whole length a yellow-gray covering, the soft palate and the pharynx are somewhat redder than usual, the neck of usual dimensions, its great blood-vessels not unusually protruding, no struma, no swollen glands. Examination of the head of the gullet with laryngoscope shows a slight thickening of the mucous membrane over the arytenoidal and santorini cartilages, nothing else abnormal; voice is clear as ordinarily, thorax well formed, the breathing regular, nothing remarkable in the percussions of the chest forward or at the right side of the back ; at the back to the left it is the same when the patient has not newly eaten, but after a full meal the percussion here is plainly shorter from the seventh to the tenth vertebra. When the esophagus is extended with air, or when he has drank wine-acid with bicarbonate, the percussion tone in these places is somewhat clearer with but little tympanitic perception. The patient has, sometimes at night, his usual pain in the cardiac region, up along the gullet, the posterior parts of the tongue and pharynx wall and the angles of the jaws. The food has begun to pass with more ease into the stomach. The cough after washing out at bedtime is ameliorated. He has now gone home, but a letter from his physician informs me that he continues to improve, though he still finds difficulty of swallowing.

The second case is that of a tinsmith, aged 62, who in his thirty-second year found difficulty in swallowing his food. This remained above the pit of the stomach and could not be got down.

The patient went once eight days without food. At other times the food, the day after eating, came up wholly unchanged most

DILATATION OF THE ESOPHAGUS FROM STRICTURE OF THE CARDIA.

frequently mixed with mucus, sometimes dissolved and offensive. He also felt, in swallowing solid food and in walking, a smarting above the pit of the stomach, and was then compelled to drink cold water to subdue the pain. He could never eat a meal without taking two to three large glasses of water. When he had drank a large quantity of cold water, the food at last entered the stomach under such powerful swallowing efforts that the veins in his forehead became swollen. Liquid food always passed down easier than solid. He got better in course of a decade, but still he was unable to get his food down unobstructed or without pain, and aliments caused a smarting in the gullet. The action of the bowels had been slow during these years. In the Winter of 1891 he received a blow in the lower part of the breast at the left side, and since then the smarting sensation has returned oftener than before. He vomited blood twice in the end of July, 1891. After this time the above distressing symptoms became worse than ever. His disposition was rendered irritable and nervous by the disease, and through economical cares. He has used ardent spirits during the latter years, but not to any considerable extent; his sleep has been generally good. He was examined on the 17th of July, 1891, and he then complained of the above-mentioned pain under the lower part of the sternum when swallowing food which, together with the drink, remained there, and of the oft-recurring vomitings which were sometimes mixed with blood. The contents of two of these were examined and they contained neither hydrochloric acid or peptones, but lactic acid. The attempt to introduce a sound did not succeed on account of the severe pain to the patient when it was pushed down into the esophagus. Nothing remarkable about the urine. Ordered liquid aliments together with pancreatin + bicarbonate $\overline{aa}$ c.g.m. 50, four times a day. The patient did not return afterward.

I did not see him again till the 26th of April, the following year, 1892. He had felt well since the year before, but was now in bed, complaining of pain in the joints, was dry in the throat and could neither get down food nor drink. His sleep was interrupted by pains about the inferior part of the sternum and in the joints, which were tender to the pressure. At the same time they were not perceptibly swollen. The patient was stiff in the neck and could only with difficulty sit up in bed. Still no considerable fever. The tongue had a gray-white covering, the fauces of a deep red. The abdomen hard to the pressure, but little yielding, evacuation only after enema. The urine free from abnormal material. The heart-tones clear, but weak. Ordered salicylate of soda in solution, of which the patient did not get down more than three grains. He began to perspire, and the next day felt better in the joints. Nourishing enema with morphine suppositories were ordered, but the enema was not retained, though the morphine gave relief from the pains, and quiet with a few nights of good sleep. As he was unable to take food, his strength shortly decreased and he died the 10th of May, 1892.

At the autopsy, which was performed the following day, the walls of the esophagus in its whole length of 26 cm., from the cricoid cartilage to the cardiac orifice, were considerably thickened. It is in its whole length widened downward, measures 6 cm. from the cricoid; cut open 7 cm. with a lumen about 2.2 cm. diameter, the thickness of the wall 0.3 cm., little further down the breadth is 8 cm., with a lumen of 2.3 cm. and wall 0.4 cm. Between its third and fourth part the breadth is 12 cm., a lumen of 3 cm., and 0.5 cm. wall from this the esophagus was suddenly constricted so that its breadth directly above and in the cardiac orifice is 2.5 cm. with a lumen 0.7—0.8 cm. in diameter with a wall thickness of 0.2 cm. (see illustration, p. 567).

The mucous membrane of the esophagus is lengthwise, strongly corrugated and thickened, measuring in the lowest quarter more than 0.1 cm. in thickness. About 1 cm. above the cardia is observed in the mucous membrane a loss of substance with cadaverish, slaty-colored surrounding and bottom, and here in the mucous membrane is a nearly circular spot of 2.5 cm. diameter, completely destroyed by ulceration, so that here is a sore with here and there projecting pointed deviations of the mucous membrane which shoots up toward the ulcer (see Fig. A). In its bottom may be seen crossing muscular fibers with the partly ulcerated thickened sub-mucus. The mucous membrane of the cardia is perfectly smooth, soft and normal. Below the cardia, on the back of the stomach, near the lesser curvature, may be observed a glandular body of the size of a bean.

The posterior and inferior parts of the lungs were found of a brittle consistency, red-brown in the section with pressure. A reddish-brown liquid exuded from the parenchyma; some of the bronchial glands were found large as peas, black and calcified. Such a gland adheres to the wall of the esophagus in a situation corresponding to the before-mentioned ulceration, and in pulling on it exhibits on the place of the ulceration, a slight but plainly marked funnel-formed depression. The stomach and other viscera give no evidence of disease, with the exception of a slight enlargement of the Peyer glands and a bloody infiltration of the large intestine. . . . . . . .

## Figures and Tables

**Figure f1:**